# Enhancing Production of Alkaline Polygalacturonate Lyase from *Bacillus subtilis* by Fed-Batch Fermentation

**DOI:** 10.1371/journal.pone.0090392

**Published:** 2014-03-06

**Authors:** Mouyong Zou, Fenfen Guo, Xuezhi Li, Jian Zhao, Yinbo Qu

**Affiliations:** State Key Laboratory of Microbial Technology, Shandong University, Ji-nan City, P.R. China; Rochester Institute of Technology, United States of America

## Abstract

Alkaline polygalacturonate lyase (PGL, EC 4.2.2.2) is an enzyme used in many industries. We developed a fed-batch fermentation process that combines the enzymatic pretreatment of the carbon source with controlling the pH of the fermentative broth to enhance the PGL production from *Bacillus subtilis* 7-3-3 to decrease the production cost. Maintaining the fermentation broth at pH 6.5 prior to feeding with ammonia and at pH 6.0 after feeding significantly improved PGL activity (743.5 U mL^−1^) compared with the control (202.5 U mL^−1^). The average PGL productivity reached 19.6 U mL^−1^ h^−1^ after 38 h of fermentation. The crude PGL was suitable for environmentally friendly ramie enzymatic degumming.

## Introduction

Ramie (*Boehmeria nivea*) fibers, the longest, strongest, and silkiest among plant fibers, are widely used in the textile industry because of their excellent properties, such as preeminent absorption, shrinkage resistance, easy dyeing, and quick drying, as well as their good mildew, bacteria, and insect resistance [1]. However, the gum (20% to 30%) in ramie fibers, which consists of pectin and hemicellulose, must first be removed before they can be applied to the textile industry [2].Large amounts of chemicals (such as NaOH) and energy are consumed during conventional chemical degumming, causing serious environmental pollution. Therefore, developing a new process using microorganisms or their enzymes for degumming has attracted wide attention [2]–[4]. Enzymatic degumming, a potential alternative to chemical degumming, is a gentle process that allows flexible operation and easy quality control, and is less damaging to fibers [5]. The main enzymes used in the process are alkaline pectinases, among which alkaline polygalacturonate lyase (PGL) is the main component [2], [4]. PGL degrades the pectin in the primary cell wall and in the middle lamella of higher plants [6], such as ramie, hemp, and flax, by catalyzing the transeliminative cleavage of polygalacturonate to produce Δ4∶5 unsaturated oligogalacturonates [7], [8]. Hence, improving PGL activity in crude enzyme promotes its application in degumming and makes it a viable option for more biochemical processes.

Submerged fermentation (SmF) is widely used for enzyme production and has been used to produce alkaline pectinase [9]. However, substrate inhibition and eventual decrease in product concentration and/or productivity often occur under high initial substrate concentration during batch fermentation [10], [11]. This condition leads to the development of the fed-batch process, which eliminates substrate inhibition and/or catabolite repression for producing metabolites and enhancing productivity. Appropriate nutrient feeding control, particularly controlled by parameters that reflect the progression of fermentation, such as pH, respiratory quotient, or dissolved oxygen (DO), ensure a successful fed-batch fermentation [12]. pH control is commonly used to optimize metabolite and enzyme production. It partially reflects the dynamic C/N ratio, which corresponds to the nutritional status of cells in the fermentation broth. Excessive pH changes affect cell growth and normal metabolism, which are important for metabolite synthesis and protein secretion. However, few studies have focused on the application of pH control for PGL production using *Bacillus* species. In addition, the existing problems in PGL production using SmF include high production costs because of the expensive reagents in synthetic media and low productivity because of the very long fermentation time [13]. Thus, strains that grow rapidly on cheap substrates through a sound SmF-based process that has high PGL yield needs to be developed to reduce the production cost.

We found that the pectinase from *B. subtilis* 7-3-3 isolated from soil effectively degrades pectin. The medium composition and culture conditions have been optimized for enzyme production using shake flasks in our laboratory, which revealed that pH is a critical factor in PGL production through the batch fermentation of *B. subtilis* 7-3-3 because it significantly affects cell growth and metabolism [14]. In this study, fed-batch fermentation was employed for pectinase production, and a pH control strategy was evaluated to further increase PGL production by *B. subtilis* 7-3-3. We also used a novel enzymatic pretreatment method that liquefies starch and reduces the detrimental effects of agitation and DO when using highly concentrated starch for feeding. The efficiency of enzymatic degumming using the PGL crude enzyme was also detected.

## Materials and Methods

### 2.1 Materials

Ramie bast fibers were friendly provided by Shandong Province Key Laboratory of Fiber Materials and Modern Textile, Qingdao University. It was confirmed that the ramie bast fiber is not a protected or endangered species. The fibers were air-dried and kept in plastic bags until use.

*B. subtilis* 7-3-3 was isolated from soil in Shandong University and preserved in the China Center for Type Culture Collection (NO: M20038).

### 2.2 Media and fermentation

The *B. subtilis* 7-3-3 inoculum was prepared by shaking the cells at 37°C and 200 rpm for 7 h in a 300 mL shake flask containing 50 mL seed culture media (glucose, 10 g L^−1^; peptone, 5 g L^−1^; yeast extract, 5 g L^−1^; NaCl, 5 g L^−1^; K_2_HPO_4_, 10 g L^−1^; MgSO_4_·7H_2_O, 0.5 g L^−1^; pH 8.0).

The cells were inoculated (10% v/v inoculum) to a 500 mL shake flask with 90 mL fermentation media (wheat bran, 54 g L^−1^; starch, 42 g L^−1^; (NH_4_)_2_SO_4_, 3 g L^−1^; MgSO_4_·7H_2_O, 2 g L^−1^; Na_2_CO_3_, 1 g L^−1^; Tween-80, 1 g L^−1^; pH 8.0) and then grown at 34°C with shaking at 200 rpm. For fed-batch fermentation in shake flasks, 10% (v/v) of the inoculum was inoculated into a 500 mL shake flask with 70 mL of the initial fermentation media (wheat bran, 47.3 g L^−1^; starch, 21.0 g L^−1^; (NH_4_)_2_SO_4_, 1.5 g L^−1^; MgSO_4_·7H_2_O, 1.0 g L^−1^; Na_2_CO_3_, 0.5 g L^−1^; Tween-80, 0.5 g L^−1^). For fermentation in a 7.5 L fermentor (Bioflo® & Celligen® 310, New Brunswick Scientific), 10% (v/v) of the inoculum was inoculated to 4 L of initial fermentation medium. The pH of the medium was maintained by feeding 10% NaOH or 25% ammonia and 30% phosphoric acid. The temperature was maintained at 34°C. The DO was maintained over 5% of air saturation via a cascaded control of agitation rate (350 rpm to 700 rpm) and aeration rate (2 L min^−1^ to 5 L min^−1^) (Silent Air Compressor, Shanghai Dynamic Industry Co., Ltd). After a corresponding phase, 1 L of feeding medium was fed to the fermentor.

### 2.3 Enzymatic pretreatment of starch in the feeding medium

The starch in the feeding medium was liquefied with mesothermal amylase digestion (25 U g^−1^ substrate) at 65°C for 30 min before it was added to the fermentor (Bioflo® & Celligen® 310, New Brunswick Scientific).

### 2.4 Enzymatic degumming of ramie bast fibers

The enzymatic degumming of ramie was as follows: 2 g of ramie bast fibers were bathed in 26 mL of Gly–NaOH buffer (pH 8.5) with different PGL concentrations at 50°C for 6 h at 150 rpm. After treatment, ramie was washed with water and dried to a constant weight at 105°C. The gum loss rate was the ratio of the weight loss of ramie bast fibers to the gum content of ramie bast fibers. The gum content of ramie bast fibers was measured according to the following method: 1 g ramie bast fibers were bathed in 30 mL of boiled 0.5 M NaOH solution for 1 h. Thereafter, NaOH solution was renewed for 2 h of boiling and then dried to constant weight at 105°C. The percent weight loss in the dry weight of ramie bast fibers corresponded to the gum content of ramie bast fibers. Inactivated PGL crude enzyme was used in the control group.

### 2.5 Analysis

#### 2.5.1 Assay of enzyme activities

Mannase activity was measured for 30 min at 50°C using konjac glucomannan (Sigma, USA) as the substrate following the method described by Hossain et al. [15]. Xylanase activity was measured for 30 min at 50°C using xylan from beechwood (X4252, Sigma, USA) as the substrate according to the method reported by Bailey et al. [16]. Filter paper activity (FPA) was measured for 60 min at 50°C using Whatman filter paper (1 cm×6 cm, 50 mg) as the substrate according to Chinese QB standard (QB2583-2003). One unit of enzyme activity was defined as the amount of enzyme that liberates 1 µmol of reducing sugar per min under the assay conditions.

Polygalacturonase (PG) was measured for 30 min at 55°C using pectin from citrus peel (P9135, Sigma, USA) as the substrate following the DNSA method [17].

The PGL activity was determined following a common method [18], except that the pH of the reaction buffer was 9.6.

#### 2.5.2 Biomass determination

The biomass was determined by assaying the level of nucleic acids in culture. The reaction mixture (containing 1 mL fermentation liquor and 1 M perchloric acid) was incubated at 100°C for 20 min and then centrifuged at 10,000 rpm for 10 min to remove unbroken cells and cell debris. The supernatant was diluted tenfold for A260 determination using a UV-visible spectrophotometer (Shimadzu, UV-2550 PC). The correlation between the resulting A260 and the cell dry weight was determined for biomass analysis in fermentation broth.

#### 2.5.3 Viscosity measurement

Medium viscosity was measured with a Brookfield viscometer LVDVE230 (Brookfield, USA).

#### 2.5.4 Determination of protein concentration

Protein concentration was determined using the Bradford method [19].

#### 2.5.5 Observation of ramie bast fibers via scanning electron microscopy (SEM)

The ramie bast fibers were degummed with PGL crude enzyme and scanned via SEM to observe the changes in surface. The ramie bast fibers were coated with platinum and then studied. Images were taken using a JEOL JSM-6700 SEM (JEOL, Japan) [20].

## Results and Discussion

### 3.1 Enhanced production of PGL in shake flasks by process optimization of fed-batch fermentation

#### 3.1.1 Effect of carbon source, salts, and (NH4)2SO4 on fermentation

**Figure 1:**
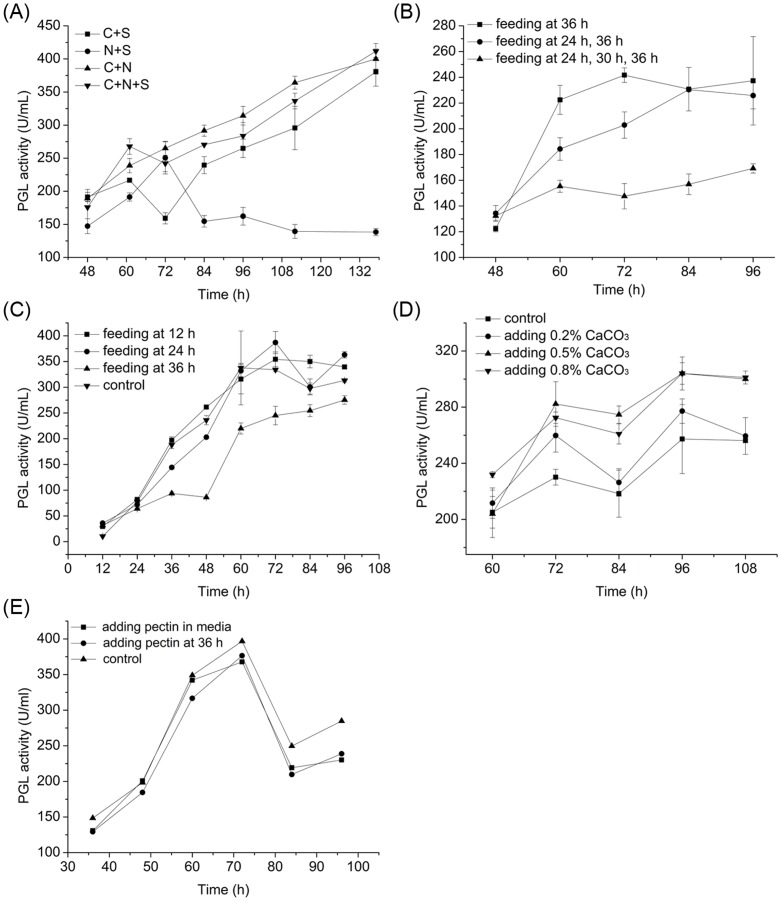
Effect of composition of the feeding medium (A), feeding frequency (B), feeding time (C), adding CaCO_3_ (pH regulated), (D) and feeding pectin (E) on PGL activity in shake flasks.

The fed-batch fermentation was first optimized in shake flasks to set up a basis for further scale up in the fermentor. We first examined the impact of the recipe of feeding media on fermentation by trying different combinations of carbon source (includes wheat bran and starch, abbreviated as C), salts (abbreviated as S), and (NH4)2SO4 (abbreviated as N). Fig. 1A shows that PGL production by the fed-batch fermentation was similar when the feeding media were C+N and C+N+S (314.3 U mL-1 for C+N and 283.7 U mL-1 for C+N+S after 96 h fermentation). Feeding N+S significantly decreases PGL activity (162.3 U mL-1 after 96 h fermentation). These results show that the carbon source is indispensable for enhancing PGL production during the late phase of fermentation. The comparison of PGL production between the fermentation fed with C+S (246.9 U mL-1) and C+N+S (283.7 U mL-1) suggested that adding nitrogen sources in the feeding media possibly improves PGL production. The addition of salts in the feeding media did not significantly affect PGL production by B. subtilis 7-3-3. The highest activity of PGL was obtained using C+N+S as feeding medium. Therefore, we used this recipe in subsequent fed-batch fermentation experiments. The same amount of feeding medium used in the fed-batch fermentation was supplemented in the batch fermentation broth prior to inoculation to compare the efficiency between batch and fed-batch fermentation.

#### 3.1.2 Effect of feeding frequency and feeding time on fermentation

Frequency of feeding is an important factor in PGL production via a fed-batch process. As shown in Fig. 1B, 72 h of fermentation fed the same amount of feeding media yielded 241.7 U mL^−1^ PGL when the system was fed only once (at 36 h), 202.9 U mL^−1^ when the system was fed twice (at 24 and 36 h), and 147.6 U mL^−1^ when the system was fed thrice (at 12, 24, and 36 h). This phenomenon indicates that more frequent feeding of smaller amounts of raw material is less effective, which suggests that smaller feedings do not meet the requirements of cell growth and metabolism, and that the cells might be starved. Thus, the energy conserved from the substrates is directed towards maintaining cellular metabolism rather than synthesizing the desired enzymes. These results suggest that single large feedings is optimal for PGL production. We further investigated the effect of feeding timing on PGL production. As shown in Fig. 1C, feeding at 24 h was optimal, with PGL activity reaching 387.0 U mL^−1^ after 72 h of fermentation.

PGL production only improved by 13.7% using the fed-batch strategy, which suggest that PGL production using the fed-batch strategy was not significantly higher than that via batch fermentation using shake flasks. However, the initial starch concentration in the medium was significantly reduced using the fed-batch process, which improved the agitation efficiency of fermentation with fermentors. Further analysis of the fermentation process of feeding at 36 h showed that the biomass was 6.4 mg mL^−1^ at 24 h and then reduced to 4.1 mg mL^−1^. Meanwhile, the average PGL productivity from 12 h to 24 h was 2.70 U mL^−1^ h^−1^, which decreased to 2.47 U mL^−1^ h^−1^ from 24 h to 36 h. Although feeding the system at 36 h improved PGL production to 245.2 U mL^−1^, which is still lower than that of the control (334.1 U mL^−1^). These results are consistent with the cell lysis starting 24 h after inoculation, which was caused by nutritional shortage in the fermentation broth.

#### 3.1.3 Effect of adding CaCO3 on PGL production

To study the effect of pH on PGL production, CaCO_3_ was added to the fermentation broth at different concentrations (0.0%, 0.2%, 0.5%, and 0.8%) to adjust pH. As shown in Fig. 1D, supplementing 0.5% CaCO_3_ resulted in the highest PGL activity (282.2 U mL^−1^) at 72 h (230.1 U mL^−1^ with 0.0% CaCO_3_, 259.8 U mL^−1^ with 0.2% CaCO_3_, and 272.5 U mL^−1^ with 0.8% CaCO_3_). We replaced CaCl_2_ with CaCO_3_ as the control to rule out the possibility that Ca^2+^ influences PGL production. Indeed, Ca^2+^ alone did not improve PGL production (data not shown). This result suggests that pH is a key factor in PGL production through fed-batch fermentation using *B. subtilis* 7-3-3.

#### 3.1.4 Effect of adding pectin on PGL production

Previous reports indicated that partially esterified (citrus) pectin or polygalacturonate in *Erwinia chrysanthemi* induces pectate lyase production [21]. Pectin was supplemented to *B. subtilis* 7-3-3–based fed-batch fermentation to determine whether purified pectin induces PGL synthesis or secretion. As shown in Fig. 1E, adding pectin did not change the process. This result suggests that adding purified pectin did not induce PGL expression in *B. subtilis* 7-3-3. This result starkly contrasts with those of PGL-producing organisms, such as *Aspergillus nidulans* [22], *Verticillium albo-atrum* [23]. *Erwinia carotovora* [24], *Erwinia chrysanthemi* [21], and *Bacillus pumilus* BK2 [25].

#### 3.1.5 Enzymatic pretreatment of the carbon source in media

**Figure 2:**
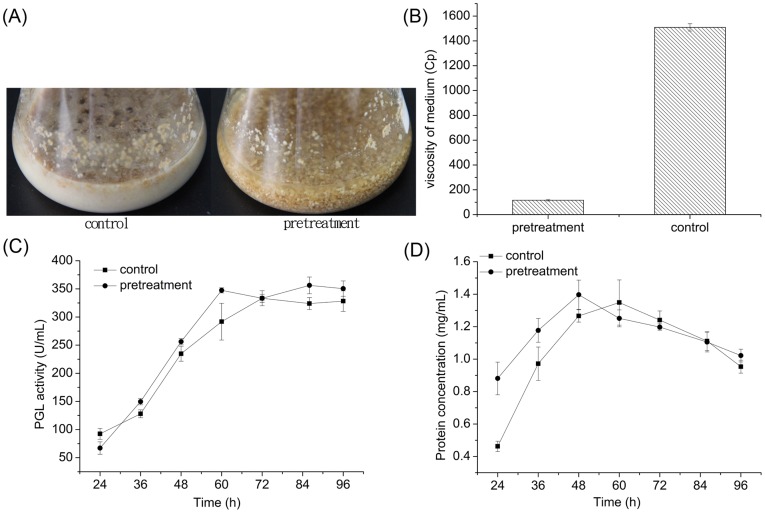
Effect of pretreating the carbon source in the feeding medium with mesotherm amylase on the state (A), viscosity of feeding medium (B), PGL activity (C), and protein concentration (D).

Although *B. subtilis* can directly use solid starch and wheat bran as carbon sources because it secretes a large amount of amylolytic enzymes into fermentation broth [26]. However, high concentrations of the gelatinized substrate (126.0 g L^−1^) in the feeding medium hindered pumping through pipelines. The high turbidity of the feeding medium could lead to further problems because it reduces the agitation efficiency, limits the mass transfer efficiency, and decreases the DO in the fermentation broth. Consequently, we used mesothermal amylase to liquefy the gelatinized substrate. As shown in Fig. 2A, the pretreatment liquefied the medium. The viscosity of the feeding medium decreased to 7.7% of the original level after the pretreatment (Fig. 2B). Fed-batch fermentation was carried out in shake flasks using the pretreated feeding medium (Fig. 2C). Using the pretreated feeding medium significantly accelerated the PGL production rate and decreased fermentation time before the PGL activity peaked (from 72 h to 60 h), which were also observed for the protein concentration of broth (Fig. 2D). This result suggests that decreasing the viscosity significantly affects the mixing and oxygen mass transfer efficiency in the fermentor under limited agitation and aeration rate [27], which are relevant to cell growth and protein production. In addition, pretreating the starch with amylase increases the saccharide concentration of the broth after feeding, thereby accelerating cell growth due to sufficient C source supply. Therefore, liquefying the gelatinized substrate with mesothermal amylase significantly enhances the PGL productivity of fed-batch fermentation using *B subtilis* 7-3-3.

In conclusion, the optimum conditions for PGL production using *B. subtilis* in shake flasks are as follows: feeding medium containing a carbon source, salts, and (NH_4_)_2_SO_4_, and single feeding at 24 h. Adding 0.5% CaCO_3_ and pretreating the carbon source further enhances PGL production.

### 3.2 Large-scale production of PGL by the fed-batch process in a 7.5 L fermentor and application of PGL in enzymatic degumming

#### 3.2.1 Production of PGL in a 7.5 L fermentor using the optimal conditions from shake flask experiments

**Figure 3:**
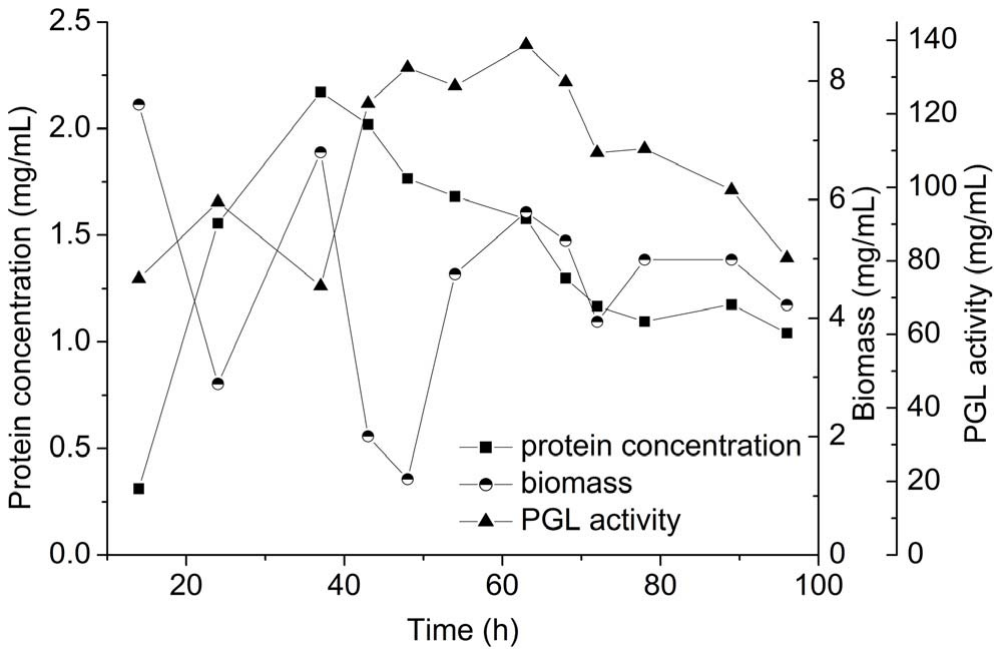
Fermentation results in 7.5 L fermentor using the optimal conditions from shake flasks.

In general, the optimal conditions in shake flask fermentation are applied to the fermentors. This is in contrast to our observation that the PGL activity obviously reduced when we used our shake flask fermentation conditions in a 7.5 L fermentor (10% NaOH was used to adjust pH). As shown in Fig. 3, the highest PGL activity (138.8 U mL^−1^) was obtained at 63 h. The biomass in the 7.5 L fermentor decreased to 2.9 mg mL^−1^ at 24 h, showing that a large number of cells had lysed. Thus, the feeding time in 7.5 L fermentors should be further optimized.

#### 3.2.2 Optimization of feeding time in 7.5 L fermentor

**Figure 4:**
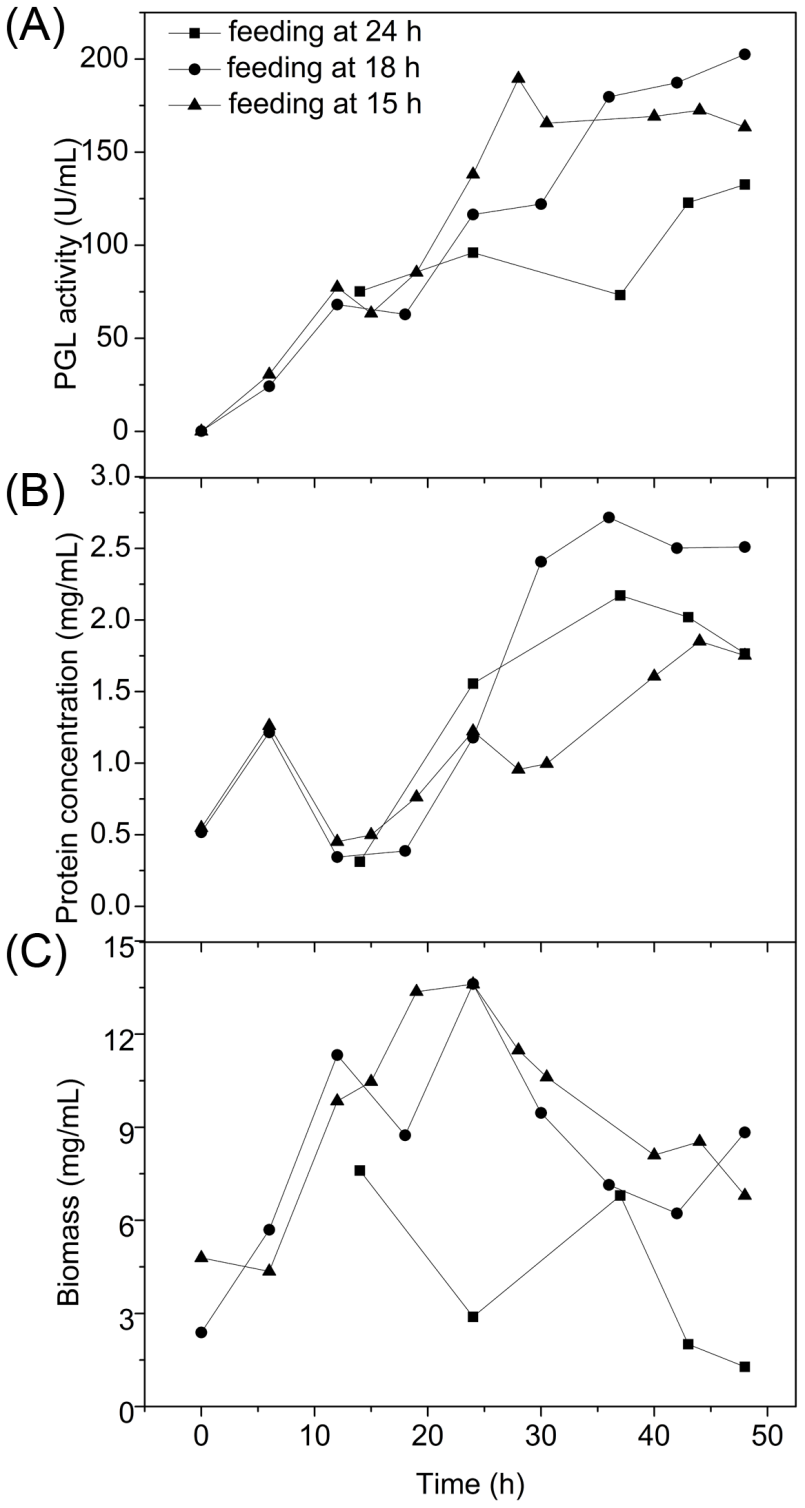
Effect of feeding time on PGL activity (A), protein concentration (B), and biomass (C) in a 7.5 L fermentor.

Feeding at an earlier time (15 h or 18 h after inoculation) in 7.5 L fermentor was shown to benefit PGL production comparing with in shake flasks (Figs. 4A and 4B). When the fermentation broth was fed at 18 h, protein concentration rapidly increased from 0 h to 6 h (from 0.52 mg mL^−1^ to 1.22 mg mL^−1^) and then rapidly reduced between 6 and 12 h (from 1.22 mg mL^−1^ to 0.35 mg mL^−1^) (Fig. 4B). The initial increase could be attributed to the induction of proteolytic and amylolytic enzymes by starch and proteins in the substrate. The following decrease could be due to the pause of protein secretion because of the fast growth of cells. The biomass apparently reduced between 12 and 18 h (from 11.33 mg mL^−1^ to 8.74 mg mL^−1^), which was reversed when the culture was fed at 18 h (Fig. 4C). This result indicates that feeding at 18 h effectively stopped cells lysis and enhanced PGL production. Feeding at 15 h had a similar consequence. In particular, the biomass did not decrease before 12 h, suggesting that feeding at 15 h was better for the production of PGL. The biomass decreased 24 h after inoculation in the cultures fed at 15 and 18 h, suggesting that a lack of nutriment could limit fermentation at this point.

#### 3.2.3 Effect of ammonia on PGL activity, biomass, and protein concentration in 7.5 L fermentor

**Figure 5:**
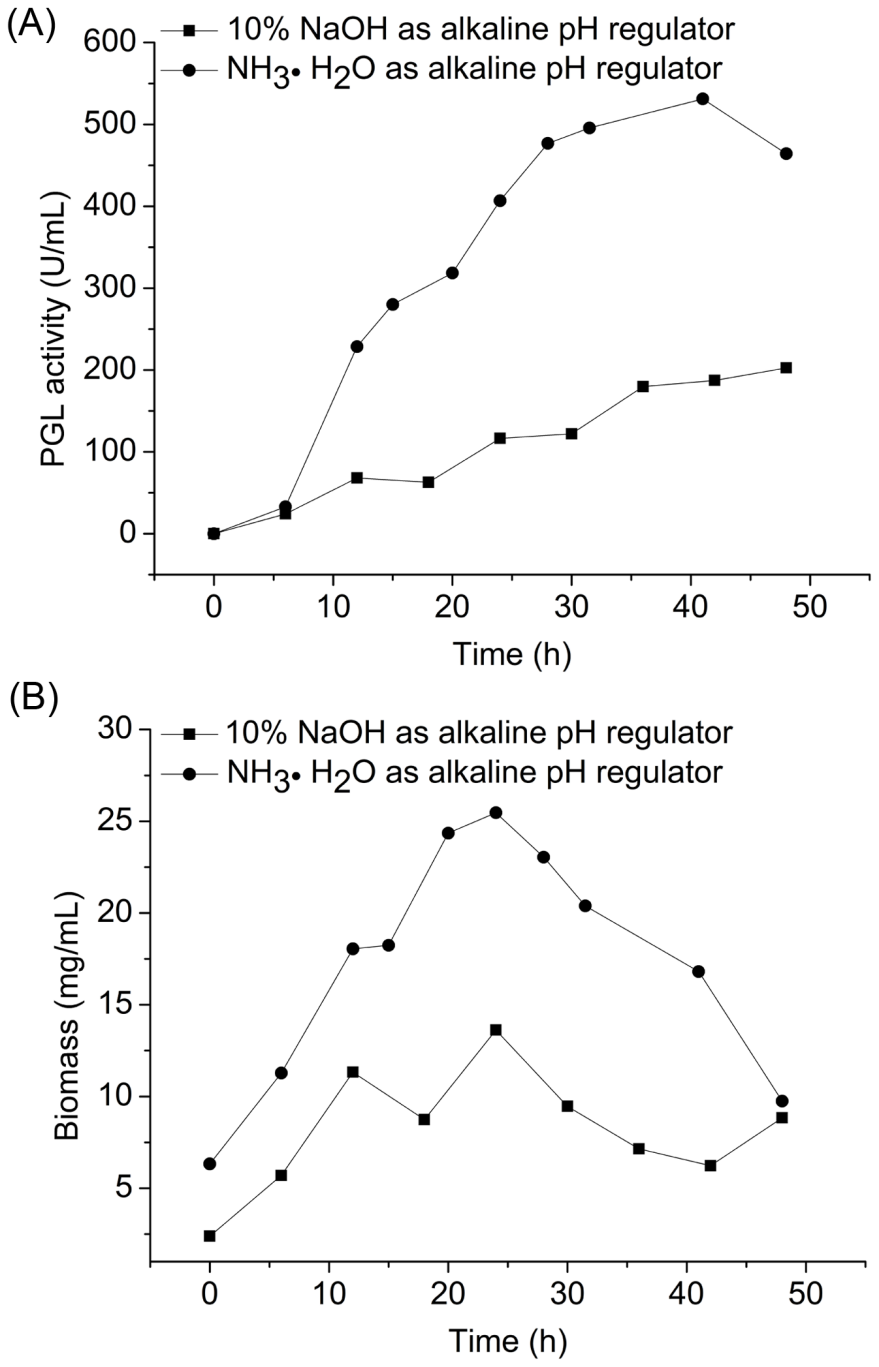
Comparison of PGL activity (A) and biomass (B) using ammonia or 10% NaOH as pH regulator in a 7.5 L fermentor.

Ammonia (NH_3_·H_2_O), which could neutralize an acidic solution and provide nitrogen source, is a commonly used alkaline pH regulator in industrial fermentation. In our experiment, a PGL activity of 531.2 U mL^−1^ was obtained when 10% NaOH was replaced with ammonia at 41 h (Fig. 5A). The highest biomass reached 25.5 mg mL^−1^ at 24 h (Fig. 5B), and the highest protein concentration reached 1.97 mg mL^−1^. The culture was fed at 15 h to avoid cell lysis. The biomass increased steadily between 12 and 20 h (Fig. 5B). These results demonstrate that ammonia is a better alkaline pH regulator than 10% NaOH. Higher PGL activity (531.2 U mL^−1^ at 41 h compared with 202.5 U mL^−1^ at 48 h using 10% NaOH) and biomass were achieved using ammonia to control pH (Fig. 5).

#### 3.2.4 Effect of pH control on PGL activity and biomass in 7.5 L fermentor

**Figure 6:**
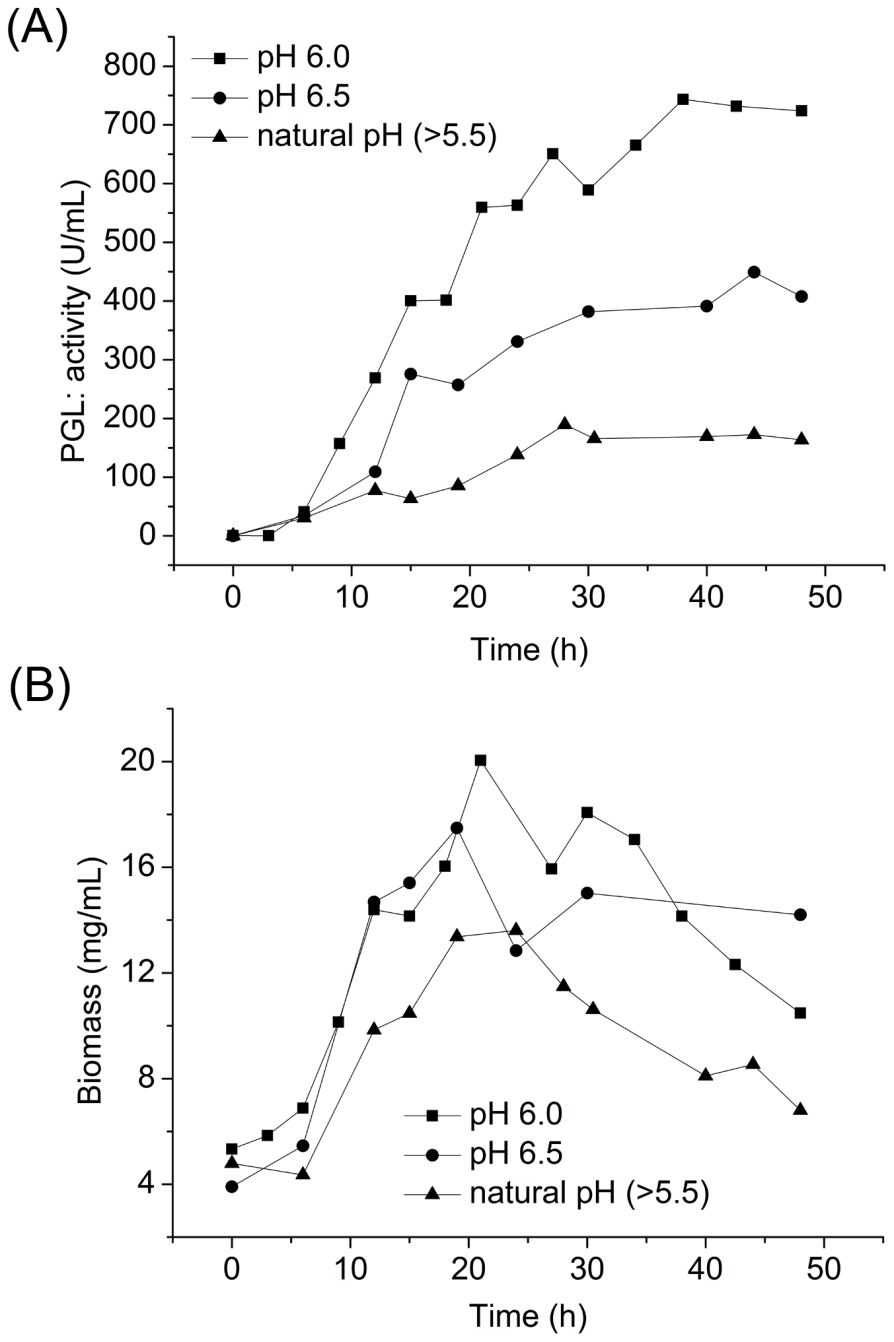
Effect of pH control after feeding on PGL activity (A) and biomass (B) in a 7.5 L fermentor.

The fermentation broth went through slow acidification prior to feeding in the fed-batch fermentation (date not shown). pH was maintained at 6.5 prior to feeding using small amounts of ammonia because of the low cell density and high DO. After feeding, the cell density rapidly increased, followed by drops in DO and pH because of the synthesis of organic acids under anaerobic or hypoxic conditions. To optimize pH control, we maintained the pH of the fermentation broth at 6.0 and at 6.5 after feeding, and compared PGL production with cultures with uncontrolled pH (natural pH, pH>5.5). PGL production was 743.5 U mL^−1^ when pH was maintained at 6.0, 449.3 U mL^−1^ when pH is maintained at 6.5, and 172.5 U mL^−1^ in natural pH, which suggests that the optimal pH for PGL production in fed-batch fermentation is 6.0 (Fig. 6A). The biomass was 20.0 mg mL^−1^ after 21 h at pH 6.0, 17.5 mg mL^−1^ after 19 h at pH 6.5, and 13.4 mg mL^−1^ after 19 h at natural pH (Fig. 6B). The biomass at pH 6.0 and at 6.5 were constantly higher than that at natural pH throughout the fermentation process, which suggests that NH_3_·H_2_O served as an additional nitrogen source for cell growth and increased cell density during fermentation. Supplementing more NH_3_·H_2_O was at pH 6.5 probably changed the C/N ratio or negatively affected cell proliferation for some unknown reasons, which reduced the PGL activity and the biomass. Combining pH control with enzymatic pretreatment of starch in the feeding medium the PGL productivity reached 19.6 U mL^−1^ h^−1^ after 38 h, which is higher than the reported 17.6 U mL^−1^ h^−1^ using *B. subtilis* strain WB43CB and 16.3 U mL^−1^ h^−1^ using *Pichia pastoris* previously (Table 1).

**Table 1:** PGL production of reported strains by optimized fermentation.

Strains	Production (UmL^−1^)	Productivity (U mL^−1^ h^−1^)	References
*Debaryomyces nepalensis*	8.73	/	[31]
*Paenibacillus polymyxa* N 10	110.42	1.53	[32]
*Bacillus subtilis* WSH02 -02	8.29	0.59	[33]
*Bacillus subtilis* strain WB43CB^a^	632.6	17.6	[34]
*Escherichia coli* ^b^	36.7	4.6	[35]
*Pichia pastoris* ^b^	1631.0	16.3	[13]
*Bacillus subtilis* 7-3-3	743.5	19.6	This work

a: Optimized strain.

b: Genetically engineered strain.

Previous studies reported that neutral proteases purified from *B. subtilis* have different activities under different pH values. Research on the properties of a neutral protease from *B. subtilis* from the Pacific Enzyme Laboratories showed that the neutral protease activity is 32% lower at pH 6.0 than at pH 6.5 [28]. Feder and Schuck observed similar results in their research on substrate hydrolysis catalyzed by *B. subtilis* neutral protease and *Bacillus thermoproteolyticus* thermolysin [29], and by Fujii on thermostable neutral proteases from *Bacillus stearothermophilus* and *B. subtilis* [30]. These observations suggest that maintaining the pH at 6.0 reduces the activity of neutral proteases and PGL hydrolysis during fermentation compared with that at pH at 6.5.

#### 3.2.5 Potential application in enzymatic degumming of ramie bast fibers

**Figure 7:**
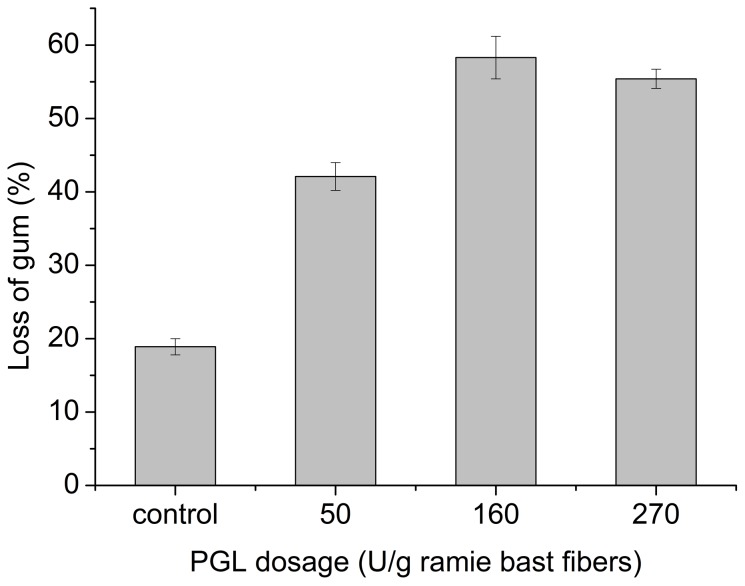
Degumming effectiveness of PGL crude enzyme in different PGL dosages.

**Figure 8:**
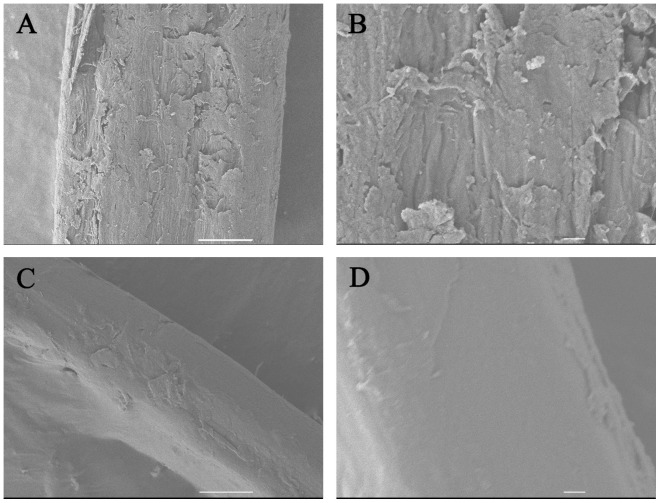
Scanning electron microscopy of ramie fibers bast before (A, B) and after treatment with crude PGL (C, D). Scale bars: 10 µm (A, C) and 1 µm (B, D).

Ramie bast fibers were degummed enzymatically to determine the activity of crude PGL produced in the 7.5 L fermentor. Approximately 60% of the gum contained in ramie bast fibers was removed through one-step enzymatic treatment at 160 U per gram ramie of PGL dosage (Fig. 7). SEM of fiber surface showed (Figs. 8A and 8B) that the PGL-treated fibers treated were flatter and smoother than the untreated fibers after removing the encrusting materials. In addition, almost no cell wall debris adhered onto the surfaces of the fibers (Figs. 8C and 8D). This result shows that PGL has good potential for degumming ramie bast fibers.

## Conclusions

Using fed-batch fermentation that combines enzymatic pretreatment of the carbon source and pH control of the fermentative broth enhances PGL production from *B. subtilis* 7-3-3. Fed-batch and enzymatic pretreatment allows the application of high concentrations of cheap substrates, which are usually used in solid-state fermentation, in SmF. The optimum pH for *B. subtilis* 7-3-3 before feeding differs from that after feeding because of the differences in physiologic states between the logarithmic and stable phases and the differences in pH dependence of neutral protease activity. Therefore, two-stage pH control was optimized to enhance PGL production. The highest PGL productivity was 19.6 U mL^−1^ h^−1^, and the crude PGL showed good potential for degumming ramie bast fibers in the pulping and textile industries.
